# A Social Media–Delivered Melanoma Prevention Program for Young Women Engaged in Frequent UV Tanning: Protocol for a Randomized Controlled Trial

**DOI:** 10.2196/56562

**Published:** 2024-03-19

**Authors:** Jerod L Stapleton, Sharon L Manne, Sherry L Pagoto, Allison Leip, Kathryn Greene, Joel J Hillhouse, Allison S Merritt, Brent J Shelton

**Affiliations:** 1 Markey Cancer Center College of Public Health University of Kentucky Lexington, KY United States; 2 Rutgers Cancer Institute of New Jersey Rutgers Robert Wood Johnson Medical School Rutgers, The State University of New Jersey New Brunswick, NJ United States; 3 Institute for Collaboration on Health, Intervention and Policy University of Connecticut Storrs, CT United States; 4 Department of Family Sciences University of Kentucky Lexington, KY United States; 5 Department of Communication Rutgers, The State University of New Jersey New Brunswick, NJ United States; 6 Department of Community and Behavioral Health East Tennessee State University Johnson City, TN United States; 7 Markey Cancer Center College of Medicine University of Kentucky Lexington, KY United States

**Keywords:** acceptance and commitment therapy, body image, dissonance-based intervention, eHealth, Facebook, indoor tanning bed, melanoma, randomized controlled trial, skin cancer, social media, sunbathing

## Abstract

**Background:**

Rates of melanoma have increased dramatically in the United States over the past 25 years, and it has become among the most prevalent cancers for young adult women. Intentional skin tanning leads to a pattern of intense and intermittent UV radiation exposure that is associated with increased risk of melanoma. Frequent tanning is most common among young women and is linked to a variety of sociocultural pressures that negatively impact body image and drive appearance control behaviors. Unfortunately, there are no established interventions designed for frequent tanners. This intervention addresses this gap with unique content informed by body image and acceptance-based interventions. The intervention is delivered using Facebook secret groups, an approach designed to support behavior change and ensure scalability.

**Objective:**

This study aims to describe the rationale and methodology of a randomized controlled trial of a melanoma prevention program targeting young women engaged in frequent indoor or outdoor UV tanning.

**Methods:**

Participants are women aged 18-25 years who report high-risk tanning (ie, at least 10 indoor tanning sessions in the past 12 months or 10 outdoor sessions in the previous summer). After recruitment and screening, participants completed a baseline survey and were randomly assigned to receive the intervention or an attention-matched control condition. Both conditions were 8-week-long Facebook groups (approximately 25 members each) with daily posting of content. Follow-up surveys are administered at 3, 8, and 18 months after baseline. The primary trial outcome is the combined number of indoor and outdoor tanning sessions reported at the 8-month follow-up. Hypothesized intervention mediators are assessed at the 3-month follow-up.

**Results:**

This project was funded by a National Cancer Institute award (R01 CA218068), and the trial procedures were approved by the University of Kentucky Institutional Review Board in February 2020. Trial recruitment and enrollment occurred in 6 waves of data collection, which started in February 2022 and closed in May 2023. The study is closed to enrollment but remains open for follow-ups, and this protocol report was prepared before data analyses. As of February 2024, all participants have completed the 8-month follow-up assessment, and data collection is scheduled to close by the end of 2024 after the collection of the 18-month follow-up.

**Conclusions:**

This trial will contribute unique knowledge to the field of skin cancer prevention, as no fully powered trials have examined the efficacy of an intervention designed for frequent indoor or outdoor tanning. The trial may also contribute evidence of the value in translating principles of body image and acceptance-based interventions into the field of skin cancer prevention and beyond. If successful, the use of the Facebook platform is intended to aid in dissemination as it provides a way to embed the intervention into individuals’ everyday routines.

**Trial Registration:**

ClinicalTrials.gov NCT03441321; https://clinicaltrials.gov/study/NCT03441321

**International Registered Report Identifier (IRRID):**

DERR1-10.2196/56562

## Introduction

### Tanning and Skin Cancer Risk

Prolonged exposure to UV radiation induces a skin tanning process in response to cellular skin DNA damage that can lead to skin cancer [1]. Excessive UV exposure through the use of artificial UV-emitting tanning beds or intense, intermittent sun exposures, like outdoor tanning (eg, sunbathing), has been linked to increased risk for all skin cancer types, including melanoma [2-6]. Excessive UV exposure is most common among young adult women and is likely driving concerning melanoma trends in the United States, including a decades-long climb in overall and site-specific melanoma incidence (eg, melanoma of the trunk) [7]. Melanoma has become among the most common cancers among women aged between 20 and 29 years [8].

Public health and policy efforts have produced an increasing number of federal regulations and state-level restrictions on access to tanning beds among minors in the past 2 decades. These efforts have helped to produce a continuing decline in the overall prevalence of tanning bed use in the United States from a peak in 2009 [9-11]. However, recent studies suggest nearly 7% of adolescents and 13% of adults still use indoor tanning in the United States each year [12], and rates of frequent, higher-risk indoor tanning remain concerning, with 24% of recent tanners reporting tanning 25 or more times in the past year in 2018 compared to 13% in 2007 [13]. Internet search results have revealed that public interest in outdoor tanning may have increased during the widespread shutdowns of tanning salons during the height of COVID-19–related restrictions [14]. National prevalence estimates of outdoor tanning are lacking because the behavior is not routinely assessed in surveillance surveys, but recent studies of young adults have found between 32% and 64% report outdoor tanning [15,16]. A recent analysis of protanning videos on the popular social media platform TikTok showed that outdoor tanning was nearly twice as likely to be portrayed as indoor tanning [17]. Overall, while indoor tanning rates have declined, intentional outdoor UV exposure or sunbathing may have increased in popularity.

Public health efforts and existing behavioral interventions have primarily focused on preventing the uptake of indoor tanning and lack attention to frequent tanning behaviors that increase the risk for melanoma development [18]. Indeed, the *Surgeon General’s Call to Action to Prevent Skin Cancer* identified a critical research gap related to an absence of interventions that target high-risk tanners and address underlying motives for tanning, including “the desire to look attractive and healthy and to conform to societal beauty standards” [19]. Further, recent trends support the need for interventions targeting frequent indoor and outdoor tanners. The proposed study addresses a gap in the melanoma prevention field by testing a skin cancer risk reduction intervention targeted to frequent indoor or outdoor tanners.

### Behavioral Determinants of Tanning Behaviors

**Figure 1:**
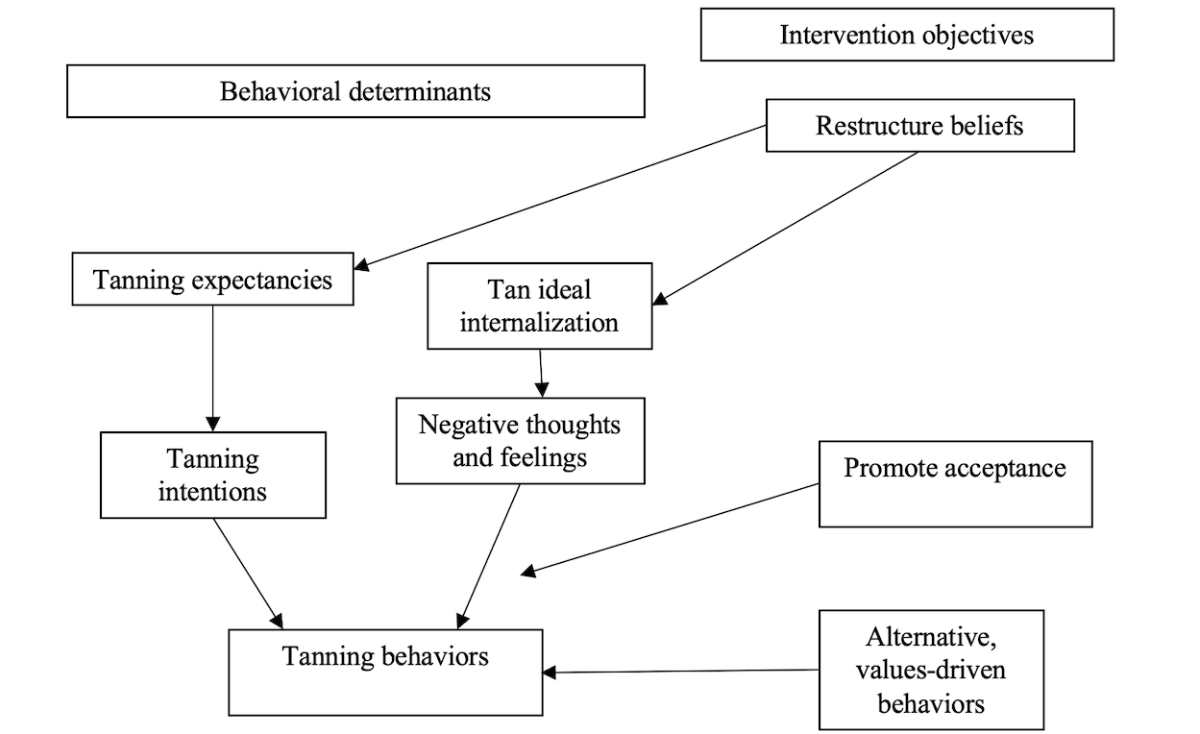
The dual process model of tanning behavioral determinants is a summary of key tanning motives identified in behavioral studies. The corresponding intervention objectives show how the intervention content is designed to target these key determinants to produce behavior change.

Our intervention is designed to address key factors from our conceptual dual-process model of tanning, which is supported by a body of behavioral research that demonstrates tanning is both an intentional, planned behavior to maintain appearance and a reactionary behavior used to avoid negative thoughts and feelings (Figure 1).

The intentional pathway is supported by studies demonstrating the role of tanning expectancies (ie, the anticipated positive and negative aspects of tanning) on tanning intentions and behavior. Tanners endorse positive aspects of tanning, including perceiving a tanned appearance as attractive, rating others with tans as more attractive than their pale counterparts, viewing indoor tanning as a convenient way to enhance appearance, and believing their peers approve of and engage in tanning [20-24]. Many young women experiment with indoor tanning in preparation for a special event, such as getting a tan before a high school prom or a wedding [25-27]. Those experimenting with tanning may begin viewing it as a regular part of their beauty routine and transition to frequent use [27]. Relaxation expectancies are also a primary motive for tanning [28], which have been reinforced by a marketing approach emphasizing a spa-like experience or fitness or health amenities for tanning [29]. This marketing may contribute to tanners’ beliefs that tanning has positive health benefits by contributing to mental wellness or self-care [28,30].

Sociocultural body image theories posit that societal influences and perceived expectations regarding appearance negatively influence body image, including how women think and feel about their bodies, and, as a result, drive them to engage in reactive behaviors they know are physically harmful for the sake of appearance enhancement and relief of body image concerns [31,32]. Research from our team and others has demonstrated that constructs from body image theories relate to indoor tanning and thus form important skin cancer risk factors among female tanners [33-38]. Tanners are likely to experience perceived pressure to look tan, which is produced in Western cultures that associate being tan as a central aspect of feminine beauty ideals and is reinforced through interactions with peers and family members. Through her buy-in and internalization to the tan ideal, a tanner views being tan as central to feeling attractive and begins actively monitoring her tan. Perceived discrepancies between her current and ideal tan will lead to dissatisfaction and negative appearance-related thoughts or feelings. In this reactive pathway, tanning represents a behavior driven by a desire to avoid such unwanted thoughts and feelings driven by routine thinking that is often flawed, negatively biased, or self-critical.

### Intervention Content

This intervention incorporates behavior change techniques and content that have been tested in our pilot tanning interventions [39-41] (see Methods section) and are designed to address both intentional and reactionary aspects of tanning (Figure 1). First, our intervention is designed to restructure key psychosocial drivers of tanning, specifically tanning-related expectancies, and body image–related beliefs underlying the internalization of a tan ideal. Participants engaged in risky behaviors may not be highly motivated to change their behavior but are likely to hold views about why they should and should not continue their risky behavior, called behavioral ambivalence [42]. Indeed, most tanners hold positive expectancies about the appearance and relaxation benefits of tanning while also reporting concerns about the appearance-damaging effects of UV exposure and perceiving advantages to reducing their tanning [43-46]. Our intervention is designed to restructure tanning expectancies by encouraging participants to reflect on underlying reasons for tanning and undesirable aspects. Such reflection has been shown to shift the balance of perceived benefits and costs of behaviors, promoting openness to behavioral change [41,42,47,48]. Drawn from body image theory–informed interventions, we also seek to restructure tanners’ body image beliefs that underlie tan internalization with content that encourages participants to critique image ideals (in this intervention, through comments in response to intervention content prompts) [49-54]. Body image interventions, including those for tanning [39,40,55], have consistently found that asking participants to consider perspectives that are critical of body image ideals and advocate for counterperspectives can change these underlying beliefs (eg, reduce perceived pressure to tan), which can ultimately reduce buy-in to these beliefs (ie, reduce internalization) and associated body image dissatisfaction. These approaches can be particularly powerful when this process occurs in a group-based setting [53,56]. Accordingly, we use Facebook groups to deliver intervention content to frequent tanners.

Although body image beliefs can be changed in brief interventions, it can be challenging because of the pervasiveness of societal messages focused on women’s appearance. Thus, the intervention is also designed to raise awareness of the impact of self-critical thoughts and negative feelings on behaviors such as tanning. The intervention messaging is consistent with the acceptance and commitment therapy (ACT) perspective that uncomfortable thoughts and feelings that accompany some deeply held beliefs cannot be controlled and attempts to do so often perpetuate self-critical thinking and maladaptive coping behaviors [57-59]. Our acceptance-based content encourages participants to embrace an acceptance mindset, defined as an increased tolerance for experiencing negative thoughts and feelings that can drive unhealthy behaviors, rather than attempting to control them through such behaviors. The intervention also includes mindfulness skill–building exercises, which are critical for enacting acceptance through increasing the ability to identify and sit with negative thoughts and feelings as they occur [57,59].

Finally, our final intervention encourages participants to focus on healthier self-care behaviors as a substitute for tanning. Consistent with ACT principles, the intervention encourages participants to consider the importance of making daily choices guided by higher-order values to ensure they spend time on the things that are truly important to them (eg, healthy lifestyle, helpful self-care, self-compassion, acceptance, and personal growth) [57,60,61]. Participants identify their core values and contrast them with the values underlying societal messages about appearance that often drive critical self-talk. Participants are then asked to consider whether their ongoing behaviors, including tanning, are consistent with their core values (eg, healthy lifestyle) and, if not, consider approaches for enacting alternative behaviors better aligned with these core values.

### Trial Objectives and Design

This randomized controlled trial (RCT) aims to examine the intervention’s efficacy versus an attention-matched control group on reducing tanning behaviors among high-risk tanners. Both interventions are delivered through the secret groups feature of Facebook, with a target group size of 25 members each. Participants were frequent tanners (indoor or outdoor) recruited from across the United States. After completing an eligibility screening and a baseline survey, participants were randomized with a 1:1 allocation ratio to each condition after finishing the baseline survey. The 8-week intervention and control groups began 1-3 weeks after baseline completion. Self-reported surveys captured tanning-related beliefs and behaviors at baseline, with follow-ups at 3, 8, and 18 months after baseline. Our primary outcome analysis will compare the tanning rates of intervention and control participants at the 8-month follow-up study. Our study is also designed to explore the impact of hypothesized mediators of intervention efficacy collected on the 3-month postbaseline survey. We hypothesize that intervention participants will report lower tanning rates at the 8-month follow-up, and these effects will be mediated by psychosocial constructs, including increasing tanning expectancies, tanning-related body image factors, and openness to changing indoor tanning. Exploratory outcomes include tanning intentions and an 18-month long-term follow-up assessment to test whether any observed intervention effects extend to the following year.

### Intervention Delivery

Intervention content is delivered within secret groups on the social media site Facebook. Participants are invited to join a Facebook group and receive twice-daily intervention content posts from a study moderator. Participants are asked to read and react to posts through the “like” feature or by commenting on them. Facebook groups provide a remotely delivered opportunity to facilitate group-based interactions within the intervention, which can facilitate stronger attitude and behavior change in disordered eating dissonance–based interventions (typically delivered within small groups) [54,56,62,63]. Further, participants in group-based social media interventions often share information and receive and provide motivation to others [64,65], which may facilitate intervention goals related to boosting positive body image and adopting healthy behaviors. Facebook remains the most popular social media site among all adults and is used by 70% of individuals on the internet who are aged between 18 and 29 years [66]. Our preliminary research found that 72% of high-risk tanners used Facebook at least once a day [67] and that Facebook-delivered tanning interventions are feasible and acceptable to our target audience.

### Choice of Control

The control condition is administered using the same procedures as the intervention, including delivery through secret Facebook groups. However, the post content differs, with posts related to nontanning health topics of interest identified in our pilot research (eg, physical activity, healthy eating, alcohol misuse prevention, stress reduction, and sleep). An attention-matched control ensures that the conditions are matched on total social media exposure, amount and type of content delivered, and intervention engagement. The design also avoids threats from demoralization among control users who expect but do not receive health content in a no-treatment control.

## Methods

### Participants

#### Study Setting

Study participants were young adult women recruited with web-based methods with reach throughout the United States. Recruitment and screening procedures were conducted primarily in partnership with Qualtrics and using Facebook advertisements. A few participants were recruited through additional methods, including posting on social media pages and recruitment flyers posted on a US college campus. Participants completed all study procedures electronically (ie, surveys and participating in Facebook groups) from their chosen location. All study enrollment, tracking, and participant engagement were conducted at the University of Kentucky. Moderating and monitoring of the Facebook groups was performed by research personnel at both the University of Kentucky and the University of Connecticut.

#### Eligibility

Eligible participants were women aged between 18 and 25 years who reported high-risk tanning (defined as using an indoor tanning bed or intentionally tanning outdoors at least 10 times in the previous 12 months) [68-70]. The study population was limited to young women due to the intervention’s emphasis on body image theory and the use of content targeted to this group. We also required that participants report regular use of Facebook (defined as self-reported use of Facebook at least 4-6 times per week in the past 4 weeks). Social media use requirements are standard in trials to ensure that participants have incorporated the chosen platform into their daily media activities and are likely to engage in the intervention [71-74].

### Procedures

#### Recruitment and Screening

Participants were primarily recruited by the internet research recruitment company Qualtrics Sample Providers using their participant panels that comply with or exceed all applicable industry standards [75]. Qualtrics emailed a randomly selected sample frame of panel participants with a study invitation, which contained a brief description of the study procedures and a brief screening eligibility assessment. Qualtrics also supplemented recruitment with web advertisements posted on various websites and platforms of their partner organizations. Additional recruitment approaches included Facebook advertisement posts with study information and links to access the screening assessment and printed flyers with QR codes to access the screener on a US college campus.

Study announcements and advertisements included a link to a 7-item brief screening assessment. Screener items assessed the eligibility criteria of gender, age, Facebook use, and past 12 months of indoor and outdoor tanning. The 2 additional health behavior questions related to the frequency of physical activity and fruit and vegetable intake were included to mask the purpose of the study. The screening survey was programmed with skip logic to identify eligible participants based on their responses. Eligible participants were provided a brief description of the study procedures immediately following the screening assessment and asked if they were interested in getting additional detailed information about the study. If they clicked yes, participants provided their contact information, which was recorded with their screening results. Screening data were reviewed for quality control to identify potentially fraudulent responses or bots. Specifically, we reviewed the pattern of responses, the total completion time, and IP addresses to identify responses that were likely from duplicate or fake respondents (including bots). Suspicious responses were removed from consideration for the study at the time of screening.

#### Study Procedures

Following screening, eligible participants who indicated their interest were emailed by study staff a link to a web-based baseline survey that also included the implied consent form. The baseline survey (and all follow-up surveys) were programmed using Research Electronic Data Capture (REDCap) survey software (Vanderbilt University) with encrypted responses collected on the University of Kentucky’s secure servers.

After completing the baseline survey, participants were randomized with a 1:1 allocation ratio to either condition. We used a permuted block randomization design to create 2 randomly shuffled assignment lists (ie, intervention or control) in each of 2 strata (ie, indoor tanning: n=500 and outdoor tanning: n=500). These lists were used to assign participants sequentially based on tanning status (within the 2 strata) following their completion of the web-based baseline survey. Following randomization, participants were invited to their assigned group using the group email feature on Facebook. As secondary options, we emailed participants to remind them to join the Facebook groups and asked participants to “friend” the moderator’s Facebook account to add them to the appropriate group manually [72].

At 3 months after baseline, each participant was invited by email to complete the first web-based follow-up survey (follow-up 1). Additional follow-ups occur at 8 months (follow-up 2) and 18 months after baseline (follow-up 3). All surveys were designed to be completed in 30 minutes. Incentives were US $30 gift cards for completing each baseline and 3- and 8-month follow-ups and US $40 for the 18-month follow-up based on similar incentive structures that have produced high retention rates in our previous indoor tanning intervention studies [76,77]. High-risk tanners often use indoor tanning throughout the year, with a peak in use in spring (ie, March-May) and outdoor tanning peaks during the summer [25,77,78]. Accordingly, our intervention was designed to be delivered during peak indoor tanning season, with the 8-month follow-up distributed near the end of the year to capture indoor tanning and cover outdoor tanning during the North American summer season. The 18-month follow-up survey will determine if intervention effects extend to tanning behaviors in the subsequent year.

### Intervention

#### Preliminary Data

Our first pilot intervention trial was designed to test the feasibility, acceptability, and preliminary efficacy of the content and messaging strategy that form the foundation of the current intervention [39]. The text-based intervention was delivered through a self-paced, single-session website. Participants were provided psychoeducation content, encouraged to reflect on their tanning behavior, and prompted to type responses to counterattitudinal perspectives to the tan ideal. We conducted an RCT in 2014 with 186 young women who reported indoor tanning at least once in the previous year assigned to receive the website or a waitlist-control condition. We found preliminary evidence of efficacy as intervention participants reported 2.29 times higher odds of abstaining from indoor tanning over a 6-week follow-up period than participants in the waitlist control (P<.05). Intervention participants also reported significantly lower intentions to tan. Participants also provided positive overall acceptability and favorability ratings of the website and content focused on body image and behavioral reflection.

Our subsequent pilot trial was to adapt this website intervention content for delivery across 8 weeks through posts to a secret Facebook group [40]. Website content was divided and delivered using 2 daily posts in the group, and participants responded to the reflection posts using the comments feature. We conducted a small, single-arm feasibility trial of the adapted intervention in a study of 17 young women who used indoor tanning beds. On average, participants viewed 92% (26/28) of all posts, reacted to (eg, “liked”) 32% (9/28), and commented on 27% (2/28) during the 8-week intervention group period. Further, 82% (14/17) of participants indicated they would recommend the intervention to a friend, and all agreed that they would continue to check the group if it were to continue. Responses to evaluation questions indicated that participants felt connected to and identified with the group, both of which are important for sustaining interest in Facebook groups [79]. Importantly, participants reported a lower number of average past-month indoor tanning sessions at the postintervention assessment (mean 0.69, SD 2.3) compared to the baseline (mean 2.31, SD 4.4; Cohen *d*=0.47).

#### Intervention Refinement

Together, our pilot work demonstrated that our intervention content and delivery platform were feasible and acceptable and had the potential to reduce tanning intentions and behavior. Before this RCT, we conducted 2 formative studies with tanners to refine our content and messaging approach further. The first was a 2018 focus group study of indoor tanners (4 focus groups [total n=20]) designed to (1) refine our planned advertising, recruitment, and enrollment strategies; (2) receive feedback on our general Facebook strategy as well as our examples of planned intervention posts; and (3) receive recommendations for how to best integrate indoor tanning as a discussion topic within the intervention. Although most participants shared that Facebook was not their most frequently used social media platform, most were active users, and they felt the Facebook group feature was uniquely valuable in connecting them with larger groups of friends or others with shared interests. Participants in all 4 groups suggested broadening the intervention purpose and post topics beyond a tanning-specific focus to promoting physical and mental wellness as part of a healthy lifestyle. Messaging related to body acceptance and female empowerment was also suggested as key desirable topics associated with a healthy lifestyle. Participants also expressed interest in content design to help them establish healthy routines and habits (eg, eating, going to the gym, and sleeping). Most participants viewed tanning as a bad habit but not necessarily as inconsistent with a healthy lifestyle. For example, tanners did not often equate tanning with other behaviors considered to be “unhealthy,” such as smoking or poor diet. However, when asked to reflect on the harms of tanning, members acknowledged that it is indeed a risky behavior and consistent with other unhealthy lifestyle choices. Finally, most participants felt they commonly encountered body image messaging and recommended we expand the discussion of body image to more broadly reflect pressures that young women experience beyond physical appearance, such as the pressure to live a “perfect” life that may cause mental stress and be inconsistent with their personal preferences.

We made several changes to the planned intervention based on this feedback. First, we refined the intervention framing, messaging, and post content to focus on promoting healthy self-care and empowerment to embrace a physical and mental wellness lifestyle. We named the intervention “empowerfulme” to reflect this focus. We also incorporated the concept of helpful self-care behaviors (ie, those with health benefits) as a contrast to unhelpful self-care behaviors, which were defined as “things we do that feel like self-care but often cause problems.” We crafted new intervention content that encouraged participants to reflect on the short- and long-term consequences of unhelpful self-care, using tanning as an example of how unhelpful self-care may be perpetuated by idealistic thinking and societal messaging. Our incorporation of messaging and exercises based on ACT approaches was also in response to the described interest in learning more about self-acceptance and living by internal values over externally imposed values.

After content refinement, we conducted a usability trial in a sample of 29 frequent tanners using the same procedures as the fully powered RCT (except for multiple follow-up assessments). We experienced a slower-than-expected rate of recruitment and enrollment that delayed enrolling a sufficient number of tanners into Facebook groups to start groups with a targeted size of 25 participants. This led to an expansion of our recruitment strategy and study description. We also examined participants’ interactions with our posts in this version and modified those that received low levels of engagement.

#### Intervention Engagement and Posting Strategy

Our intervention is administered within a study-specific “secret” Facebook group with membership and content limited to invited group members. Facebook groups ran for 8 weeks and contained 25 members each. Given that ideal intervention length and group size have not been empirically established and differ based on intervention content and objectives [74], we modeled our intervention on our pilot Facebook study. Our intervention included strategies found to boost participant involvement and group engagement in Facebook group–delivered health interventions for young adults [40,71,72,80,81]. We provided clear expectations, including checking the group study account at least once a day and “icebreaker” activities to increase comfort with commenting. We also used a young woman as the intervention moderator; all posts came from this account. Under the research team’s guidance, the moderator commented or reacted to various posts (ie, liking them) to reinforce participant activity and encourage engagement.

Intervention content was delivered through posts made by the group moderator twice daily for 8 weeks, for a total of 112 posts. Each week addressed a thematic topic introduced in the first weekly post (Monday morning), with corresponding goal-setting and monitoring activities throughout the week (Table 1). Other posts were designed with activities and prompts to address change objectives, reinforce intervention messaging, or promote group engagement [40]. We also structured the content and timing of our posts to maximize engagement. Posts were written in a conversational tone and designed to take less than 3 minutes to read and comment on. A graphic designer enhanced the visual appeal of our posts and included relevant photos and videos [82]. We included polls and prompts in our posts for participants to share tips and successes with others [74,83].

**Table 1:** A description of the types, frequency, purpose, and content of the posts used for intervention and control group content.

Post type	Weekly frequency	Purpose and content
Goals	3	Participants set a goal related to the weekly topic (Monday evening), completed monitoring or check-in related to progress (Thursday morning), and reported back about goal progress (Sunday).
Reflection and discussions	5	To restructure key expectancies and beliefs with content that is discussion-based and designed to encourage reflection on one’s ongoing behavior or speaking out against idealistic perspectives.
Reinforcement messages	2-3	Posts drawn from popular sources and designed to reinforce intervention messaging. Unlike other “active ingredient” posts, content may change between waves to accommodate ongoing events or provide recent and relevant articles.
Sharing content	2-3	Memes, inspirational quotes, and humorous posts intended to promote engagement and provide positive messaging.

#### Intervention Content

Our intervention strategy and content were developed through a mapping process [84] that included (1) identifying specific behavioral determinants (ie, factors that lead to tanning behavior) to target for change (described previously); (2) developing specific performance objectives (POs) to specify the changes in these targets that must occur to produce the overall behavioral outcome of reducing tanning; and (3) applying behavior change theory and techniques to produce intervention content to meet the POs.

POs 1-3 (Table 2) are designed to restructure tanning expectancies and body image beliefs that drive tanning intentions and idealistic thinking. Initial intervention content is framed within the context of considering that “self-care” behaviors can be either helpful (eg, exercise) or unhelpful (ie, they may provide some immediate benefit but have costs in the short- or long-run). Participants are asked to reflect on their self-care and reflect on their view of whether tanning is helpful or unhelpful self-care (POs 1 and 2). Participants are asked to consider the balance of benefits and problems with various forms of self-care and consider plans to increase helpful and reduce unhelpful self-care. Tanning-specific content encourages participants to reflect on underlying reasons and undesirable aspects of tanning to restructure beliefs by shifting the balance of pros and cons and thus promoting openness to behavioral change [41,42,47,48].

PO 3 also seeks to restructure beliefs by incorporating counterattitudinal advocacy techniques that effectively alter risky body image beliefs [49-54]. This approach has the person speak out against and question commonly held idealistic thoughts. If done effectively, the alternative perspectives considered during these exercises will conflict with idealistic thinking, producing psychological discomfort (ie, cognitive dissonance) [63]. The person is then motivated to seek psychological relief by altering their original unhealthy beliefs to be more consistent with the healthier perspective being advocated. Creating dissonance is optimized when participants share counter perspectives in group-based settings, which leads to more robust attitude and behavior change [53,56]. Accordingly, the intervention approach is to solicit counterattitudinal comments as responses to our Facebook group posts.

POs 4 and 5 are guided by the ACT concept of promoting acceptance. It can be difficult to completely change and mitigate the impact of strongly ingrained beliefs, such as those underlying idealistic thinking. ACT defines fused beliefs as routine thinking that can be self-critical and may lead to unwanted thoughts and feelings that drive habitual behaviors that are often unhealthy in an attempt to control these difficult experiences [57-59]. The intervention provides psychoeducational content and messaging that thoughts and feelings cannot be controlled and attempts to control them can create additional problems and perpetuate self-critical thinking. The intervention messages emphasize the value of willingness to experience negative thoughts and feelings by using mindfulness skills [57-59].

Finally, PO 6 encourages participants to consider the importance of making daily choices guided by higher-order values [57,60,61]. The intervention content encourages participants to consider the importance of broad values (eg, healthy lifestyle, helpful self-care, self-compassion, acceptance, and personal growth) and consider their alignment with societal messages and expectations promoting tanning. Participants identify their core values and contrast them with the values underlying societal messages about “perfection” in appearance and other aspects that often drive self-critical thinking. They are also asked to consider whether their ongoing behaviors are consistent with their core values and engage in goal-setting for enacting behaviors aligned with them [57,60,61].

**Table 2:** The intervention performance objectives (POs) that guided the content of corresponding intervention posts.

POs	Intervention content
PO 1: engage in helpful self-care behaviors	1a. Describe helpful self-care behaviors 1b. Identify benefits of helpful self-care behaviors 1c. Identify and enact plans for increasing helpful self-care behaviors
PO 2: reduce unhelpful self-care behaviors (with tanning as an example)	2a. Identify unhealthy forms of self-care, including tanning 2b. Describe how tanning is not aligned with helpful self-care. Describe problems with tanning. 2c. Identify and enact plans to reduce unhelpful self-care
PO 3: reduce buy-in to appearance messages about ideal women	3a. Critique societal expectations for women that lead to negative affect and unhealthy behaviors like tanning 3b. Describe the messages underlying these expectations and their sources 3c. Speak out against the problems caused by self-critical appearance values
PO 4: increase acceptance and willingness to experience uncomfortable feelings	4a. Identify problems with trying to control thoughts and feelings through behaviors like tanning 4b. Understand the concept of acceptance 4c. Express a commitment to adopting an acceptance mindset
PO 5: practice mindful acceptance	5a. Enact general skills for self-observation (ie, mindfulness skills) 5b. Practice identifying self-critical beliefs and related feelings and sitting with them
PO 6: engage in behaviors that are consistent with personal growth values	6a. Describe the most important personal growth values 6b. Evaluate alignment of current behaviors with growth values 6c. Identify and enact plans to ensure values-consistent behavior

### Measures

#### Self-Report Survey Items

Our primary outcomes are self-reports of indoor and outdoor tanning at the 8-month follow-up (Table 3). We chose items that are expert-recommended [85] and commonly used in tanning intervention trials [39,41,77]. Outcomes have open-ended response options to increase reporting accuracy and have been shown to be reliable compared to daily diary reports of behavior assessed over the same period [77,86]. Mediators and other measures are also listed in Table 3 and include measures adapted for tanning and validated in our previous research [38].

**Table 3:** The trial measures captured with self-report surveys.

Construct		Description of measures
**Primary outcomes (measured at 8-month follow-up)**		
	Indoor tanning	Open-ended reporting of the number of times participants used a tanning bed or booth in the past 8 months [85]
	Outdoor tanning	Open-ended reporting of the number of times spent outdoor tanning in the most recent summer (eg, between June 1, 2023, and August 31, 2023) [86]
**Secondary outcomes**		
	Tanning intentions	Intentions to use indoor tanning or spend time outdoor tanning (2 separate questions) in the next 12 months [39,41,77]
	Long-term tanning	Indoor and outdoor tanning outcomes assessed at the 18-month follow-up
**Primary mediators (measured at 3-month follow-up)**		
	Tanning expectancies	Beliefs related to tanning benefits (eg, tanning is attractive and relaxing) and risks (eg, appearance damage and skin cancer risk), tanning attitudes, and temptations to tan [87,88]
	Tanning-specific body-image factors	Items adapted from the Objectified Body Consciousness Scale to assess actively monitoring and comparing one’s tans with others [38,89] Internalization of the thin and tan ideal [21,35,38,88] Appearance conversations with friends [90] Items adapted from the Multidimensional Body Self-Relations Questionnaire to reflect tan dissatisfaction [38,91]
	Openness to changing tanning	Perceived difficulty in stopping indoor or outdoor tanning (2 separate questions) [92] The Readiness to Change Questionnaire adapted to tanning behavior [93]
**Exploratory mediators**		
	Values and values-consistent living	Valued Living Questionnaire [94] Valuing Questionnaire [95]
	General body image acceptance	The Acceptance and Action Questionnaire II [96] Beliefs About Appearance Scale [97]
	Perceptions and use of tanning alternatives	For both sunless tanning products and spray-tanning, past 12 use, intention, attitudes, and self-efficacy [77,85]
	Mindfulness	Mindful Attention Awareness Scale [98]
**Covariates**		
	Demographics	Age, education, race, ethnicity, urban, rural, and SESa
	Sun protection habits	Typical use of sun protection (eg, shade and sunscreen) [99]
	Use of social media	Frequency of use of popular social media sites and integration of Facebook within social behaviors and daily routines [67,100]
	Melanoma risk factors	Natural hair color, eye color, skin type, skin reactivity to the sun, and history of sunburns [101]
	Depression, anxiety, and stress	The Depression Anxiety Stress Scale-10 [102]
	Other health behaviors	Alcohol use [103] Cigarette use [104] Physical activity [105] Sedentary behavior [106] Sleep [107] Diet [108]

^a^SES: socio-economic status.

#### Facebook Engagement

The 3-month follow-up survey contained several Facebook-specific evaluation items, including perceived connectedness with a group, identification with posts and other group members, enjoyment, and ease of participation [79,109,110]. Items from the Audience Engagement Scale were used to measure key aspects of engagement with intervention content, including personal involvement (ie, was information judged to be personally relevant) and personal reflection (ie, was the knowledge acquired used to reevaluate personal conduct) [111]. Software (GRYTICS [112]) was also used to capture objective measures of participants’ engagement, including comments and reactions to our posts or comments from others within our posts.

### Statistical Analysis

#### Power

The primary outcome will be a sum of the 8-month indoor tanning outcome and the number of outdoor tanning sessions in the summer. One of the only intervention trials to focus on high-risk tanners was an in-person counseling intervention that produced significantly lower tanning rates among intervention participants at a 3-month follow-up [113]. This analysis suggested means for indoor tanning sessions of 4.40 (SD 7.74) for the intervention group and 11.78 (SD 13.03) for controls for a between-group difference of 7.38, which corresponded to a moderate to large size effect (0.69). Because our intervention is less intensive than a counseling intervention, we powered our study based on the scenario that the treatment effect will be at least 60% of the counseling study (corresponding to an effect size of 0.41). In our usability trial baseline data, the mean number of previous 3-month measures of indoor tanning and past summer outdoor tanning was 22.5 (SD 22.9; our means will likely be higher in the trial since the past 8-month indoor tanning will be used). Assuming similar means in our trial control group, an intervention effect of 0.41 would produce a mean of 13.1 (SD 22.9) tanning sessions in the intervention group, a difference of 9.4 total tanning sessions. Assuming these estimates from our follow-up means (ie, tanning control: mean 22.5, SD 22.9; tanning intervention, mean 9.4, SD 22.9), we will achieve 80% power with at least 186 participants (93 in each group). We anticipated a 70% follow-up rate at the primary 8-month outcome, which led us to target the enrollment of 266 participants to test the effects adequately.

#### Primary Outcome Analyses

Using intention-to-treat analyses, 2-sided tests for the effect of treatment will be conducted at the 0.05 level. If less than 10% (26/266) of outcomes are missing, we will consider data to be missing at random and apply multiple imputations. If missing data are more than 10% (27/266), we will examine patterns of missing data, including comparing demographics for those with and without missing outcomes. If missing data are not random, we will consider alternative missing data approaches. We will also conduct sensitivity analyses by first comparing baseline characteristics by condition and modifying the analyses to adjust for differences.

Multilevel models (eg, random coefficient) will test the primary study hypothesis that participants who received the intervention will report less total tanning behavior at 8-month follow-up compared to those who received the control. The level 1 model represents individuals nested within Facebook groups (the level 2 model). In particular, the effect of the intervention will be considered a random effect that may vary by block (ie, Facebook group). Thus, the model for analysis will use the following form as a starting point: *Y_ij_* = *β*_0_*_j_* + *β*_1_*_j_T* + *ε_ij_*, where *Y_ij_* represents the number of tanning sessions used for the *i*th individual within the *j*th block, *T* is an indicator for whether the individual was randomized to the treatment or control. As such, the analysis accounts for (by including the random intercept *β*_0_*_j_*) random differences between blocks (eg, previous tanning behavior) and allows the effect of the intervention to vary by block (with *β*_1_*_j_*). If necessary, sensitivity analyses may control for individual-level covariates and appropriate transformations (ie, square root), or additional analysis methods will be considered if the normality assumption is strongly violated.

#### Mediation Analyses

Mediator analysis will be used to assess whether participant attitudinal factors (level 1 variables), including tanning expectancies, tanning-related body image factors and change perceptions, mediate the effect of the intervention. In particular, for each potential mediator, the 4 component steps [114] will be examined through regression models similar to those described above. The contribution of the mediated effect will be assessed directly using the approach recommended by Kenny and colleagues [115]. For multilevel models, we will calculate the product of the 2 pieces of the mediating path (from intervention group assignment to the mediating variable and from the mediating variable to the outcome). Bootstrap procedures will be used to formally test for the significance of the mediating pathway.

### Ethical Considerations

The University of Kentucky Institutional Review Board reviewed and approved all study procedures before data collection (review number 56153 2019). A web-based informed consent form was included as the first page of the baseline survey, and it described the study procedures, privacy, confidentiality, risks, benefits, and data protection. Participants provided implied consent by agreeing to the consent form and starting the survey. All survey data were collected with a deidentified approach, using a unique participant identifier in place of a name or other personal information. The engagement data downloaded from Facebook were deidentified before storage or analysis by replacing participant names with their unique identifier. Our consent form also provided a description of the certificate of confidentiality provided by the National Institutes of Health as part of their funding support. Participants were emailed Amazon gift card incentives in the amount of US $30 for completing each of the baseline, 3-month, and 8-month surveys and US $40 for the 18-month follow-up.

## Results

This trial was funded by the National Cancer Institute (R01 CA218068) in July 2017 and approved by the University of Kentucky Institutional Review Board in February 2020. Trial recruitment and enrollment occurred in 6 waves of data collection, starting in February 2022 and closing in May 2023. The study is closed to enrollment but remains open for follow-up. This protocol report was prepared before data analyses. As of February 2024, all participants have completed the 8-month follow-up assessment, and data collection is scheduled to close by the end of 2024 after the collection of the 18-month follow-up.

## Discussion

Frequent tanning has been linked to an exponential increase in melanoma risk, but there are no established interventions targeting this group. This is the first study to test an intervention for frequent tanners in a fully powered trial. The prevailing approach to tanning interventions has been to raise awareness about the health risks as well as the appearance-damaging effects of tanning (eg, premature skin aging and wrinkling) [77,116-120]. These interventions have been delivered as educational-based interventions in various formats, with some incorporating UV facial photographs showing existing skin damage in combination with health risk information. Several trials have demonstrated the efficacy of these appearance-focused interventions when tested among typical tanners (eg, participants who have tanned at least once). However, the efficacy of this approach is likely to be limited among more frequent tanners [121] who generally report knowledge of and perceived susceptibility to the appearance risks of tanning but believe the immediate benefits outweigh the longer-term risks [20,122,123].

Beyond their primary focus on occasional tanners, appearance-focused interventions do not address key motives that are likely driving frequent tanning, including sociocultural influences, body image, and tanning dissatisfaction. This intervention approach represents an extension of the previous intervention literature by attempting to change core motives and influences that make tanning “worth the risks” among frequent tanners as well as incorporating key elements of an acceptance-based intervention. A small number of studies have demonstrated the potential value of using dissonance-based, body image–focused intervention strategies in melanoma prevention efforts [39,40,55,124]. This trial extends this work by (1) testing a fully powered skin cancer prevention program delivered entirely through social media; (2) delivering the intervention to both frequent indoor tanners and sunbathers; (3) broadening the intervention content focus beyond body image ideals to also challenging general expectations of how young women should look, think, and act; and (4) translating concepts from ACT principles to create acceptance-based content that encourage participants to accept that negative thoughts and feelings will happen and consider the importance of making daily choices that are guided by higher-order values.

Existing indoor tanning interventions have not been widely disseminated. The use of social media to deliver intervention content embeds the intervention into a platform that has become ingrained in the lives of our target young adult population and is a preferred method for exchanging information and communicating with peers [125]. A Facebook-delivered intervention can be integrated into individuals’ existing, routine social media habits and avoids the burden of requiring users to find and use a stand-alone, unfamiliar website. The impact of this work lies in the potential for dissemination in a variety of contexts, given the use of a familiar and freely available intervention platform. This study will produce an intervention guide and content library of posts that could be implemented with minimal costs and staffing. Further, the intervention allows for social connection and self-expression among participants, which are key reasons people use social media [109]. These intervention features, along with the focus on positive body image, may increase the interest of potential intervention participants and thus lead to a more impactful intervention.

### Limitations

Potential limitations to using Facebook include the fact that the nascent social media literature lacks firmly established, empirically based guidelines for effective practice. Thus, several of our choices (eg, content, length, and group size) are based on our experiences in pilot studies and available best practice guidelines. An alternative to the proposed Facebook approach would be retaining the previously tested website intervention. Websites have advantages, including increased control over content, easier user tracking, and the ability to deliver more in-depth content. However, website interventions have restrictive barriers for the target population, including the need to direct them to an unfamiliar website and a possible lack of interest in engaging on the website for any substantive period of time (as noted in our focus group research). The group discussions critical to the success of dissonance-based intervention are more easily implemented on the freely available Facebook setting but are more difficult to achieve with a website. There are also limitations in using Facebook to deliver content across several weeks. Developers of Facebook group support interventions for smoking cessation have raised concerns about the “empty room phenomenon,” which describes the hesitancy of some social media users to engage with strangers [64]. We have engaged in several formative user-centered studies to inform the messaging and content of our intervention, but low engagement can occur in social media–delivered interventions. It is possible that users could alter the topic of discussion in ways not conducive to indoor tanning prevention or make unfavorable comments. We attempt to prevent this behavior by providing general guidelines for posting, daily monitoring, and following an established intervention manual for moderators.

### Conclusions

This trial will contribute unique knowledge to the field of skin cancer prevention, as no fully powered trials have examined the efficacy of an intervention designed for frequent indoor or outdoor tanning. Our intervention builds from and meaningfully extends existing skin cancer interventions and incorporates innovative elements from body image and acceptance-based interventions in its content and delivery. If efficacious, findings can inform best practices in skin cancer prevention and provide evidence of the value of translating principles from body image and acceptance-based interventions to target other behavioral contexts. The use of Facebook groups allows group-based interactions among participants, which can facilitate stronger changes in attitudes and behaviors and provide a platform to embed the intervention into individuals’ everyday routines.

## Appendix

Multimedia Appendix 1 Peer-review report by the National Cancer Institute (National Institutes of Health).

## Abbreviations

ACT: acceptance and commitment therapy
PO: performance objective
RCT: randomized controlled trial

## Data Availability

The data sets generated during this study are not publicly available due to the data sharing plan that was approved as part of our funded application and the certificate of confidentiality provided by the National Institutes of Health. Deidentified data sets are available from the corresponding author on reasonable request following the issuance of our final project funding report.
