# Supplementary material for: A Social Media–Delivered Melanoma Prevention Program for Young Women Engaged in Frequent UV Tanning: Protocol for a Randomized Controlled Trial
Source: JMIR Res Protoc. 2024 Mar 19;13:e56562. doi: 10.2196/56562 (PMC10988380; doi:10.2196/56562)
Supplement: Multimedia Appendix 1 [file resprot_v13i1e56562_app1.pdf]

1R01CA218068-01 Stapleton, Jerod

INCLUSION OF MINORITIES PLAN UNACCEPTABLE
EARLY STAGE INVESTIGATOR
NEW INVESTIGATOR
SCIENTIFIC REVIEW OFFICER'S NOTES

RESUME AND SUMMARY OF DISCUSSION: This well written application seeks support to conduct a Facebook based intervention testing body image theory and persuasive techniques to reduce indoor tanning among young women at high risk of skin cancer. This high impact works addresses a highly significant public health challenge as skin cancer rates continue to increase and these rates are directly relatable to sun tanning. Reviewers noted many strengths that led to their assessment of high impact in this outstanding study. These strengths include an exceptional scientific premise supported by outstanding pilot data, an exceptional and impressively productive Principal Investigator and research team additionally enhanced by the collaboration with consultant Dr. Sherry Pagoto, an exceptional research environment, an outstandingly innovative focus on a Facebook delivered intervention targeted to a uniquely vulnerable group of high risk frequent tanning young women with a novel focus on reducing the pressures to tan by promoting positive body image rather than focusing on information about the risks of tanning. Furthermore, reviewers were enthusiastic about the outstanding scientific premise characterized by excellent qualitative and quantitative pilot data and evidence presented to both describe the lack of effective strategies to reduce tanning behavior and the promise here to apply body image theory to inform this novel intervention. Reviewers deemed the scientific rigor strong to outstanding in the inclusion of a rigorous randomized controlled design, a cost effective, readily disseminable intervention if successful, inclusion of mediational analyses to confirm the mechanisms by which the intervention exerts its potential impact and a long follow up with proposed outcomes tested at 18 months. Some minor and moderate concerns were discussed which reduced the score slightly: the self-report nature of tanning behavior might be susceptible to bias, a minority view questioned the control group used here and noted concern with the generalizability of the sample, others disagreed. Concerns were noted regarding the statistical analysis plan. In sum, this is an outstanding study that offers high impact to reduce indoor tanning and ultimately skin cancer among an ideal target group of young women who are high risk tanners.

DESCRIPTION (provided by applicant): The number of U.S. adults treated annually for melanoma, the most deadly form of skin cancer, has nearly doubled in the past 15 years and the incidence rate is projected to again double by 2030. This growth is partly attributed to the popularity of artificial ultraviolet-emitting indoor tanning beds. Despite recognition by numerous national and international health organizations as being carcinogenic to humans, nearly 10 million Americans use indoor tanning beds each year. An estimated 1 in 10 of all new U.S. cases of melanoma and 400,000 annual cases of non-melanoma skin cancer are directly attributable to indoor tanning. Most concerning, nearly 1 in 5 young adult white females engage in high-risk indoor tanning, defined as using indoor tanning beds at least ten times a year, which is associated with a substantially increased risk of melanoma. As indoor tanning gained popularity among young women over the past two decades, melanoma has recently become one of the most common cancers among this population. The 2014 Surgeon General's Call to Action to Prevent Skin Cancer identified a critical research gap related to an absence of interventions that target high risk indoor tanners and address underlying motives for tanning, including “the desire to look attractive and healthy and to conform to societal beauty standards”. The purpose of this application is to implement and test an intervention designed to encourage indoor tanning cessation among high-risk tanners. The intervention is unique in using persuasive techniques and content to reduce perceived pressure to be tan, reduce the value placed on tanning, and promote positive body image rather than focusing on information about the risks of tanning. The intervention will be delivered via the social media site Facebook through the “secret groups” feature. The use of Facebook groups will allow group-based interactions among participants, which can facilitate stronger changes in attitudes and behaviors,

and provides a platform to embed the intervention into individuals' normal routines. The first proposed aim is to refine the existing intervention content from our preliminary intervention studies with user-generated feedback. The second aim is to conduct a randomized controlled trial of the intervention in a sample of 400 young women engaged in high-risk tanning. Our primary hypothesis is that participants who receive the intervention will report less indoor tanning at a 6-month follow-up compared to those who participate in a control Facebook group. Our third aim will examine hypothesized psychosocial mediators of the intervention effects. The intervention has strong potential for cost-effective, widespread dissemination and targets a group at high-risk for future cancer development. If effective, the intervention has the potential to significantly reduce the growing burden of melanoma and other skin cancers.

PUBLIC HEALTH RELEVANCE: The use of artificial ultraviolet indoor tanning beds is associated with an increased risk of skin cancer, including the deadly melanoma. This project involves developing and testing a novel behavioral intervention delivered via the social media site Facebook that is designed to reduce high-risk indoor tanning behaviors among young adult women. If effective, the intervention may be widely disseminated and has the potential to help reverse concerning melanoma trends observed among young women.

CRITIQUES: The written critiques of individual reviewers are provided in essentially unedited form below. These critiques were prepared prior to the meeting and may not have been revised afterwards. The "RESUME AND SUMMARY OF DISCUSSION" above summarizes the final opinions of the committee.

CRITIQUE 1:

Significance: 5
Investigator(s): 1
Innovation: 3
Approach: 6
Environment: 1

Overall Impact: A Facebook-based intervention, using persuasive techniques to reduce indoor tanning among young women is proposed, based on the sound premise that young women are at high risk of developing skin cancer as a result of their tanning behavior, but also unlikely to change their behavior because of an underlying desire to conform to societal beauty and image standards. The problem being addressed is a significant one, and the target group, although limited by age, tanning frequency and gender is an appropriately high risk group (so the significance is not diminished because of the fairly narrow focus). While the scientific approach to an RCT is rigorous, the RCT itself may not include the appropriate control group to actually address the significance of the underlying problem, and as a result may not fit in with the overall scientific premise. The method of selection of participants to the trial makes generalizability hard to determine (further affecting the overall impact). While the focus on Facebook and it is “group” features is innovative, the analysis plan does not capitalize on the group effects. Some aspects of the scientific premise are not well described or justified (particularly the link with behaviors occurring in eating disorders). While there is some excellent pilot data provided, the intervention and control materials have not yet been developed, which results in the possibility of a failed Aim 1 rendering the remaining aims difficult to complete. These are moderate weaknesses but the excellent investigative team should be able to address them all.

1. Significance:

Strengths

- Indoor tanning is a strong risk factor for skin cancer, and not only is tanning highly prevalent in young women (the target group), but it is also a highly preventable exposure.
- Skin cancer is an important public health problem, and probably increasing in the target age group.

Weaknesses

- While the target population is certainly of the “Facebook generation”, it is unclear how reliance on Facebook affects generalizability and internal validity (that is, are we only getting those participants most likely to be affected by interventions because they respond to this kind of FB-based intervention – e.g. do not hang out with their friends in person: those people may well be more affected by tanning norms than others, and more likely to be affected by the messaging being delivered.
- The key scientific premise is based on the statement that “The application of behavioral research linking body image to tanning suggests interventions for high-risk tanners should target body image-related constructs” – but the argument that “body image” in other behavioral research is synonymous with pressure to tan, tan valuation and body dissatisfaction with respect to skin tone or tan is fairly weak.
- Again with respect to the main premise, much is made of the link to eating disorders and behaviors – but eating disorders and tanning are very different entities. The case for why the theory behind eating disorder behavior and tanning behaviors should be similar is not well made.
- While FB-disseminated interventions could have the potential to be easily and widely adopted, it is not clear how “platform dependent” they are – is the next generation replacement for FB going to have group-based features that are key to this intervention?
- It is not entirely clear that a comparison of two different FB-based arms will answer the main question being posed – there should perhaps be a non-FB arm to provide context in the use of social media as the delivery method. This application is actually answering the question “is tanning information on FB better than general health information on FB?” rather than whether or not FB (and/or a group approach) is better than other methods of preventing indoor tanning.

2. Investigator(s):

Strengths

- The Principal Investigator is a new investigator with an excellent level of productivity and a clear research agenda aligned with a well described career path in understanding the determinants of tanning behaviors, and most of his work has been in the population this application is targeting (indoor tanners).
- The investigative team is well rounded and covers all the required skills to ensure a project like this is successful.
- The investigative team has a track record of working with one another.

Weaknesses

- None noted.

3. Innovation:

Strengths

- There is innovation in the skin cancer field in the use of persuasive techniques and content to reduce perceived pressure to be tan, reduce the value placed on tanning, and promote positive body image rather than focusing on information about the risks of tanning.

Weaknesses

- Facebook-based interventions are not particularly innovative
- There is a missed opportunity to capitalize on the group-based innovation in the overall theoretical model and the proposed analysis.

4. Approach:

Strengths

- Strong pilot data on the efficacy and acceptability of the main components of the intervention

Weaknesses

- While the focus work identified that lots of people use FB (particularly to form groups), there is little evidence provided that they would like to have their health interventions delivered that way (in fact precisely the opposite might be true!), nor that the materials to be used in this intervention are acceptable (because they haven't been developed yet).
- While the approach allows for the use of group-based interactions, the proposed analysis plan and specific aims do not make use of this data (e.g. with a multilevel analysis or comparison).
- As noted in Significance, there is insufficient consideration given to the generalizability and internal validity of the Approach.
- While the pilot data on FB users notes that the tanning bed use was lower, it is not clear if that is a seasonal effect (tanners tend to use tanning beds more in Spring, less in Summer – as noted in the application) – that seems likely given that the participants appear to be college students.
- While some pilot data have been provided, the bulk of the early work in this application appears to be to develop the materials that will be used. There is ample evidence that the team will be able to do that, but it seems like that work should be done prior to proposing the RCT – that is, the actual intervention well defined and refined, and in particular the "control" materials which do not even have to be well-suited to tanning prevention (they could really be just about any health-related material). There is the possibility that the effort in Aim 1 will fail (participants might hate all the materials, and/or they will need to be dumbed down so much that the key intervention constructs are lost).
- Statements like "selectively procure relevant content from other Facebook groups or pages" are quite vague – it is not clear how those selections will be made, and what the selection process is.
- The use of Qualtrics for sample selection is an unknown quantity when it comes to generalizability, even among frequent internet users – who is selected and who is not? (These questions might be impossible to answer with the proposed recruitment strategy)

5. Environment:

Strengths

- The combination of institutions involved in this application bring more than sufficient expertise, resources and history of innovative science to the application.

Weaknesses

- None noted.

Protections for Human Subjects:
Acceptable Risks and/or Adequate Protections
Data and Safety Monitoring Plan (Applicable for Clinical Trials Only):
Acceptable

Inclusion of Women, Minorities and Children:
- Sex/Gender: Distribution justified scientifically
- Race/Ethnicity: Distribution justified scientifically but the minority representation is not clear.
- For NIH-Defined Phase III trials, Plans for valid design and analysis:
- Inclusion/Exclusion of Children under 18: Excluding ages <18; justified scientifically
- Tanning bed use is banned in children.

Vertebrate Animals:
Not Applicable (No Vertebrate Animals)

Biohazards:
Not Applicable (No Biohazards)

Budget and Period of Support:
Recommended budget modifications or possible overlap identified:
- 50 percent effort for the Principal Investigator throughout the project is not well justified.

CRITIQUE 2:

Significance: 1
Investigator(s): 1
Innovation: 2
Approach: 2
Environment: 1

Overall Impact: This potentially high impact application is responsive to the 2014 Surgeon General's Call to Action to Prevent Skin Cancer and addresses indoor tanning, a strong risk factor for melanoma. Melanoma rates have doubled over the past 15 years and are forecasted to double again over the next 15; therefore, this is considered an important line of research. This application is put forth by a new, early stage investigator who has already conducted a substantial body of work in this area and this application builds on this momentum. A careful research plan is proposed whereby investigators will conduct additional formative research to hone the intervention so that it delivers messages that are dissonance-based and well integrated into a Facebook delivery channel. Then, the team will assess the impact of the intervention vs. an attention control condition on tanning rates at 6-months (and again at 18-months) and also will conduct mediational analyses to confirm the mechanisms by which the intervention exerts its potential impact. The team that is amassed for this initiative is exceptionally strong, as is the environment. Moreover, the research that is proposed is considered scientifically

rigorous and meritorious. Perhaps, the only concern is the need for additional formative work and full development of the intervention, particularly since this team has already had ample opportunity to complete this work.

1. Significance:

Strengths

- This project is aimed at indoor tanning, a significant risk factor for melanoma for which the risk has doubled in the past 15 years and which is projected to double again by 2030. Despite this increase in risk, this is a relatively understudied area of investigation.
- His application is responsive to the 2014 Surgeon General's *Call to Action to Prevent Skin Cancer.*
- The project is aimed at developing and testing an intervention which will be immediately incorporated into the UMass Center for mHealth and Social Media. If efficacy is proven, it also will be incorporated into the Indoor, Tan-free, Skin Smart Campus Initiative at UMass. Importantly, because it will use Facebook, it is designed to be scalable beyond this particular market long term.

Weaknesses

- None noted.

2. Investigator(s):

Strengths

- This application is put forward by a new, early stage investigator, Jerod Stapleton, PhD who has already made substantive contribution to the biobehavioral literature as it relates to risky behaviors among young adults. To date, he has published 22 articles on indoor tanning, which is the focus of the proposed study. Moreover, he has been the recipient of both an R03 and K07 which are on the same topic area from the NCI and is considered well-qualified to lead the proposed investigation.
- The research team also is strengthened by the efforts of the following investigators: 1) Kathryn Greene, Professor of Communication at Rutgers, who is an expert in health communication and decision making among adolescents; 2) Joel Hillhouse, Professor of Community and Behavioral Health at East Tennessee Status University who is nationally renowned for his expertise in indoor tanning and sun safety; 3) Sharon Manne, Professor of Medicine and Section Chief of Population Sciences at the Cancer Institute of New Jersey who is known more for her work in dyadic interventions, but has also published in the area of indoor tanning; 4) Pamela Ohman-Strickland, Associate Professor of Statistics at Rutgers; and 5) Sherry Pagoto, Associate Professor at the University of Massachusetts Medical School and cofounder of the UMass Center for mHealth and Social Media, and also an expert in skin cancer prevention. This is an incredibly strong research team and many of these investigators have mentored Dr. Stapleton previously.
- The research team has collaborated on several common papers thus there is ample evidence of an established and successful working relationship.

Weaknesses

- None noted.

3. Innovation:

Strengths

- The team proposes to adapt some of the science that exists regarding body image theory and will construct dissonance-based messages to change perceptions regarding tanning.
- The intervention will focus on a population that is at particular risk of melanoma because of their indoor tanning practices and will use a social networking based approach to deliver the intervention.

Weaknesses

- Facebook-based interventions are not necessarily novel.

4. Approach:

Strengths

- This application builds on a considerable body of work and preliminary studies by the investigative team. Ample justification is provided that dissonance-based interventions offer a promising means of crafting health messages and that Facebook is a viable channel.
- A well-orchestrated schedule of formative research is proposed to hone the intervention and a careful discussion is provided to justify the timing, group size, timing of prompts, participant burden, etc.
- A rigorous control group is proposed for the efficacy trial (AIM 2)
- A mediational analysis is proposed for AIM 3 and will provide additional data to thoroughly understand the mechanisms by which the intervention may work.
- Ample discussion and justification is provided to defend scientific rigor.
- A particular strength of the efficacy trial is that it not only will test for efficacy at 6-month follow-up, but also will assess differential tanning rates between study arms at 18-months as well.

Weaknesses

- While the intervention is already developed, it still needs to be adapted prior to testing. Therefore, a substantial amount of development is still necessary before it can be tested.

5. Environment:

Strengths

- This project relies on the incredibly rich environment provided by the collaboration between CINJ and Rutgers, UMass, and ETSU. Moreover, a successful means of identifying potential participants using Qualtrics, Inc. a resource that many investigators at CINJ have used successfully is proposed.

Weaknesses

- None noted.

Protections for Human Subjects:

Acceptable Risks and/or Adequate Protections

Data and Safety Monitoring Plan (Applicable for Clinical Trials Only):

Acceptable

Inclusion of Women, Minorities and Children:

- Sex/Gender: Distribution justified scientifically
- Race/Ethnicity: Distribution justified scientifically
- For NIH-Defined Phase III trials, Plans for valid design and analysis: Not applicable
- Inclusion/Exclusion of Children under 18: Excluding ages <18; justified scientifically

Vertebrate Animals:
Not Applicable (No Vertebrate Animals)

Biohazards:
Not Applicable (No Biohazards)

Budget and Period of Support:
Recommended budget modifications or possible overlap identified:

- A travel allowance of $4K for scientific conferences is justifiable in the two later years, but seems excessive for the initial years of this project.

CRITIQUE 3:

Significance: 1
Investigator(s): 1
Innovation: 1
Approach: 3
Environment: 1

Overall Impact: This is a very well-written application that will test the application of an intervention based on body image theory to indoor tanning behavior among high-risk tanners. The scientific premise is quite strong, with evidence presented for the need to address high-risk tanning behavior (i.e., frequent indoor tanning), the lack of effective strategies, and the promise for applying body image theory to inform a novel intervention. The investigative team is outstanding, and the environment is excellent. The scientific rigor of the approach is extremely high, with careful thought put into all aspects of study design. There are a few minor concerns, outlined below, but overall the approach is very carefully considered.

1. Significance:
Strengths

- Indoor tanning has been shown to be a risk factor for skin cancer.
- High-risk tanners, who engage in indoor tanning more frequently, are resistant to educational interventions that focus on the health consequences of tanning.
- Drawing from work in the area of eating disorders, there is the potential to impact the behavior of high-risk tanners by addressing factors related to body image.
- Initial work applying body image theory to tanning behavior suggests that this may be an effective approach to reduce indoor tanning behavior.

Weaknesses

- None noted

2. Investigator(s):

Strengths

- The investigative team is outstanding, and includes experts in indoor tanning, melanoma prevention, health communication, biostatistics, and social media for health behavior change.
- Each of the investigators has collaborated to some extent with the Principal Investigator, and many have worked with each other before.
- The Principal Investigator is a young investigator with an impressive early track record for publications and a demonstrated familiarity with indoor tanning.

Weaknesses

- None noted

3. Innovation:

Strengths

- The utilization of a theoretical framework (body image theory) that has been used successfully to modify behavior related to eating disorders to address tanning behavior is innovative.
- Focusing on high-risk tanners is unique and delivers the intervention to those who may benefit from it the most.
- Using Facebook private groups as a medium to reach intervention targets holds promise to magnify the impact of the intervention, which gains salience from the group exposure.

Weaknesses

- None noted

4. Approach:

Strengths

- Relevant pilot work has been conducted by members of the proposed investigative team and supports the likelihood of the intervention having an impact on the participants. In particular, an analysis of 823 women using latent profile analysis indicates that women favorable tanning beliefs and elevated body concerns are more likely to be high-risk tanners.
- A pilot study of 186 women found high engagement with a website-based intervention that is similar to the proposed Facebook-based intervention and showed initial indications of behavioral change in the desired direction.
- The design of the intervention is informed by qualitative data obtained from 5 focus groups.
- A feasibility trial in which 17 indoor tanners used a private Facebook group to receive tanning-relevant content indicated high engagement with the intervention and suggests that members of the target population will adhere to the recommended intervention regimen.
- Plans for the refinement of the Facebook-based intervention are carefully laid out and include content refinement, formative user testing, and usability testing prior to rolling out the complete intervention.

- Timing of the study is planned to be maximally effective, given the seasonality of indoor tanning.
- Recruitment of study participants will occur using a commercial company, meaning that participants will not be geographically limited to one homogeneous area. One of the investigators has experience recruiting through this company, which increases confidence in the process.
- The intervention content is planned and corresponds with framework of body image theory.
- The inclusion of questions about other health behaviors (alcohol, physical activity, stress) to mask the focus on tanning is a very good element to the study. The investigators might want to consider adding smoking, and making sure that the number of questions asked about tanning does not still make the focus evident.
- Planned mediation analyses will greatly enrich the study results.

Weaknesses

- It is suggested that “participants resistant to stopping tanning will be encouraged to consider sunless tanning options”. Although not stated, it is assumed that this reference refers to, for example, spray-on tan products. This approach seems to run counter to the body image approach, wherein the purpose is to convince participants that they do not need to look tan to be beautiful. Moreover, this strategy appears to suggest some tailoring of advice, which is not otherwise addressed in the intervention design. If the intervention will be adaptive based on participants’ level of resistance to the message, this facet should be explicitly addressed. (minor).
- Given the focus that will be apparent in the intervention group on tanning, there is a potential for participants to give inaccurate reports of their indoor tanning frequency at the end of the study because of social desirability bias. It might be useful to include some measures of social desirability to check for this possibility. (minor)
- The reliance on self-report of frequency of tanning sessions reduces the internal validity of the study. Evidence that the items used to elicit self-reports have been validated in other contexts alleviates this concern somewhat, and it is difficult to see how else the behavior might be assessed, but it might be worth considering some sort of ongoing assessment via the facebook medium, rather than only a pre- and post- test. (minor)

5. Environment:

Strengths

- The respective research environments for each of the investigative team members are excellent, and the Principal Investigator appears to have very strong institutional support for research.

Weaknesses

- None noted.

Protections for Human Subjects:

Acceptable Risks and/or Adequate Protections

- Risks to participants are minimal, and a plan is in pace to minimize risks.

Data and Safety Monitoring Plan (Applicable for Clinical Trials Only):

Acceptable

- A DSMP is presented and is adequate.

Inclusion of Women, Minorities and Children:

- Sex/Gender: Distribution justified scientifically
- Race/Ethnicity: Distribution justified scientifically
- For NIH-Defined Phase III trials, Plans for valid design and analysis:
- Inclusion/Exclusion of Children under 18: Excluding ages <18; justified scientifically
- The study will focus on young women adults, because this is the population most at risk for melanoma associated with indoor tanning.

Vertebrate Animals:
Not Applicable (No Vertebrate Animals)

Biohazards:
Not Applicable (No Biohazards)

Budget and Period of Support:
Recommended as Requested

THE FOLLOWING SECTIONS WERE PREPARED BY THE SCIENTIFIC REVIEW OFFICER TO SUMMARIZE THE OUTCOME OF DISCUSSIONS OF THE REVIEW COMMITTEE, OR REVIEWERS' WRITTEN CRITIQUES, ON THE FOLLOWING ISSUES:

PROTECTION OF HUMAN SUBJECTS: ACCEPTABLE

INCLUSION OF WOMEN PLAN: ACCEPTABLE

INCLUSION OF MINORITIES PLAN: UNACCEPTABLE. The inclusion of minorities is unclear and should be clarified and possibly oversampled.

INCLUSION OF CHILDREN PLAN: ACCEPTABLE

COMMITTEE BUDGET RECOMMENDATIONS: The budget was recommended as requested.

SCIENTIFIC REVIEW OFFICER'S NOTES: The NIH special practice for new investigator R01 applications reviewed in the Center for Scientific Review study sections applies to this application. Resubmission (amended -A1) R01 applications from new investigators may be submitted on a special receipt date for review in the very next review cycle. See this notice in the NIH Guide for Grants and Contracts for more details: http://grants.nih.gov/grants/guide/notice-files/NOT-OD-11-057.html.

You should contact the NIH program officer whose name is shown in the upper left hand corner of page one of this Summary Statement for information about whether this application may be fundable or whether you will need to submit an amended application. The program officer can also help you decide whether the changes and improvements necessary to address the weaknesses noted in the reviewers' critiques could be accomplished in the relatively short time available. You are also strongly advised to seek input from mentors, your Department chair, etc.

If you choose to submit a resubmission application for the next review cycle under this policy for new investigators, your amended application must be received at NIH no later than Monday, April 10, 2017.

You may, of course, choose to take more time to resubmit your application. If so, you should prepare the resubmission for the normal dates for amended applications as specified in this table: http://grants1.nih.gov/grants/funding/submissionschedule.htm.

Footnotes for 1 R01 CA218068-01; PI Name: Stapleton, Jerod

NIH has modified its policy regarding the receipt of resubmissions (amended applications). See Guide Notice NOT-OD-14-074 at http://grants.nih.gov/grants/guide/notice-files/NOT-OD-14-074.html. The impact/priority score is calculated after discussion of an application by averaging the overall scores (1-9) given by all voting reviewers on the committee and multiplying by 10. The criterion scores are submitted prior to the meeting by the individual reviewers assigned to an application, and are not discussed specifically at the review meeting or calculated into the overall impact score. Some applications also receive a percentile ranking. For details on the review process, see http://grants.nih.gov/grants/peer_review_process.htm#scoring.
